# PW02-008 - TRAPS in the real world: an international registry

**DOI:** 10.1186/1546-0096-11-S1-A148

**Published:** 2013-11-08

**Authors:** HJ Lachmann, R Papa, K Minden, L Obici, L Cantarini, J Frenkel, J Anton, I Koné-Paut, M Cattalini, B Bader-Meunier, N Ruperto, A Martini, I Touitou, P Woo, M Gattorno

**Affiliations:** 1 PRINTO, Eurotraps/Eurofever Projects, Genoa, Italy

## Introduction

TRAPS is an autosomal dominant disease due to mutations in the TNFRSF1A gene.

## Objectives

To analyze the clinical features of TRAPS and genotype/phenotype correlations of TRAPS patients enrolled in the Eurofever/Eurotraps registry.

## Methods

The Eurofever Project (agreement n 2007332, EAHC) is aimed to build a common web-based registry for all Autoinflammatory diseases in collaboration with the Eurotraps Project (FP7, HEALTH-F2-2008-200923). A web-based registry collecting demographic and clinical information on Autoinflammatory diseases is available in the member area of the PRINTO web-site.

## Results

158 TRAPS pts were enrolled from November 2009 to June 2012. analyzed. The majority of pts were adults (105, 67%). The median age at disease onset and at disease diagnosis were 4.1 (range 0.2-63 years) and 25.9 (0-77) years, respectively. The median delay of diagnosis was 10.3 years, with a rate of delay 10 times higher in adult patients. Patients were enrolled by 18 centers in 11 countries, with 18 different countries of origin. 145 (91%) were European Caucasians, 4 Arabs, 3 black Africans, 2 Asiatics, 2 Ashkenazi Jewish, 2 had a mixed origin. 58 pts (36.7%) carried high-penetrant mutations (T50M or involving cysteine residues), 30 pts (19%) other missense mutations previously associated to TRAPS phenotype. Low-penetrant or hypomorphic variants, such as R92Q and P46L, were found in 53 and 5 pts, respectively. 12 pts carried mutations with uncertain association with TRAPS phenotype. High penetrant mutations were observed in 50/123 (40%) pts with pediatric onset and in 8/35 pts (22.9%) with an adult onset (p < 0.001). The prevalence of patients carrying low-penetrant or hypomorphyc mutations were two time higher in the adult onset (48%) compared with pediatric onset (25.2%) (p < 0.001).

Mean duration of fever episodes was 10.6 days with mean number of 6.6 episodes/year. In one third of the cohort a mean duration of fever episodes was shorter than 1 week. The most frequent symptoms accompanying attacks were fever, limb pain (arthalgia, myalgia), abdominal pain and a variety of skin rashes. The prevalence of a number of clinical manifestations significantly differ among the age of onset. Pediatric patients displayed an increased prevalence of enlarged cervical lymph nodes abdominal pain and periorbital edema in respect to patients with an adult onset. Adult onset was characterized by an higher prevalence of respiratory symptoms (chest pain, persistent cough), serositis and polyarthritis. The most common long term complication was amyloidosis, that was observed in 16 (10%) adult patients. The mean age at onset of amyloidosis was 43 years (range 20-77). Patients who developed AA amyloidosis had significantly longer disease duration than those who have not (39 years versus 19.4 years; p=0.001).

## Conclusion

TRAPS has a wide geographical distribution and can be observed in different ethnicities. Short episodes (less than 1 week) are observed in 30% of patients. The presentation of the disease in adulthood significantly differs from that observed in pediatric age.

## Disclosure of interest

None declared.

